# Visions for a JACIE Quality Management System 4.0

**DOI:** 10.1038/s41409-021-01467-8

**Published:** 2021-09-29

**Authors:** John A. Snowden, Eoin McGrath, Kim Orchard, Nicolaus Kröger, Anna Sureda, Alois Gratwohl

**Affiliations:** 1 Department of Haematology, Sheffield Teaching Hospitals NHS Foundation Trust, Sheffield, UK; 2 EBMT Executive Office, Barcelona, Spain; 3 Department of Haematology, University Hospital Southampton, NHS Foundation Trust, Southampton, UK; 4 Department of Stem Cell Transplantation, University Hospital Eppendorf, Hamburg, Germany; 5 Catalan Institute of Oncology, Barcelona, Spain; 6 Hematology, Medical Faculty, University of Basel, Basel, Switzerland

**Keywords:** Health services, Stem cells

## Abstract

Quality management has been part of hematopoietic stem cell transplantation (HSCT) from the very beginning. It evolved step-wise from open data exchange up to the introduction of the FACT/JACIE-based quality management system (QMS) 2 decades ago. This formal step has eased cooperation, and improved outcome for patients. Today’s expansion of cellular and targeted therapies and new drugs, and the regulatory requirements for advanced therapeutic medicinal products have touched the limits of the current system. Based on the Medicine 4.0 concept, the next step should integrate novel views of QMS. The old definition “Best Quality Transplant” will be replaced by “Optimal Treatment,” and encompass the entire health care journey. “Best outcome” will refer to overall survival, quality of life and costs, with or without HSCT, and will be compatible with all requirements by competent authorities. Decisions will be based on high-level evidence, supported by real-time digitized data collection, data analysis, incorporated into artificial-intelligence systems. To reach this goal, EBMT/JACIE will be challenged to start the process by further fostering harmonization within and between organizations at institutional, national, and European levels. Acceleration in information technology and modifications to working practices during the pandemic should facilitate this development to the next stage.

## Introduction

Today’s concept of hematopoietic stem cell transplantation (HSCT) is based on the seminal experiments of Jacobson and Lorenz in the middle of last century: spleen or bone marrow cells from donor animals could repopulate hematopoiesis in rodents after lethal total body irradiation of the recipient [1, 2]. The idea rapidly gained traction with HSCT viewed as a potential tool in the advent of a nuclear accident or nuclear war by some, and as a novel approach to treat leukemia by others. For the latter, total body irradiation was seen as an instrument to eradicate malignant cells, healthy donor bone marrow as replacement, and the combination of both as a provider of cure for patients with leukemia [3–6]. The pioneering first clinical transplants in patients with advanced leukemia by the later Nobel Prize winner E.D. Thomas gave proof of principle [7]; they paved the way to today’s global success of HSCT [8–11].

Quality management systems (QMS) were not considered in the early period of HSCT, but the critical concepts were informally in existence. They were instrumental that bone marrow transplantation continued, despite the fact that none of the first five patients survived, and in two patients only donor hematopoiesis was documented [7]. Admittedly, the early investigators did profit from the “window of opportunity” out of fear of the atomic bomb [12]. But they initiated the basic quality concepts: responsibility, transparency, reproducibility, accountability, and open data exchange. The simple fact “it can be done” sufficed as formal evidence. More important, the early pioneers did strive for continuous improvement, and learned from their errors.

The present era—following on from the pandemic, where there have been many modifications to health care practice, and information technology (IT) systems—is an appropriate time to look back at the evolution of HSCT and, moving forwards, of the quality management process in tandem, and at the different steps in their respective time frames. Viewing the development of data collection, data analyses, benchmarking, study conduct, hence, in quality management helps to become aware of the achievements as well as to understand some substantial deficiencies in concurrent treatment approaches. It is also timely to position this retrospective into the framework of “Medicine 4.0,” a term put in context with the terminology of the” Industrial Revolution 4.0” in 2015 by the World Economic Forum [12–18]. It describes the four steps of industrial processes from the mechanization (1.0), electrification (2.0), automation (3.0) to today’s digitization (4.0). “Technology 4.0” integrates development, production, and delivery in a complex digitalized and continuously updated automated system, and evolves as a continuous process of improvement, embedded in a formal QMS. In a similar though simpler way, we can look at the different steps in the evolution of The Joint Accreditation Committee of ISCT and EBMT (JACIE).

## “QMS 1.0”: the beginning in the 1980s with “standardized reporting”

The first comprehensive series by IBMTR in 1970 summarized the then available experience of clinical bone marrow transplantation worldwide; 3 out of more than 250 patients survived long term [19]. The publication provoked deep concerns regarding bone marrow transplantation, not least because of the perceived poor outcomes. It was the transparent communication and the continuous drive for improvement that kept the trust of patients and referring physicians in the nascent bone marrow transplant community.

Several factors further fostered the development: the recognition of HLA-matching between donor and recipient, the development of intensive induction regimens for patients with acute leukemia that allowed transplantation in early phase of the disease, and the advent of modern immunosuppression [5, 7, 9]. A first wave of transplants began. Out of need for correct comparisons, members of the CIBMTR, EBMT, and the Seattle transplant team joined forces. They proposed a model for standardized reporting and data presentation. Outcome was defined by four main endpoints: overall survival, disease-free survival, transplant-related (or non-relapse-) mortality, and relapse incidence [20]. The former should be visualized as descending, the latter as ascending Kaplan–Meier curves. They proposed a three tiered structure, based on the basic patient-, disease-, donor-, and transplant-related factors. Minimal-Essential Data MED-A (EBMT) or Transplant-Essential Data TED-A (CIBMTR) described the minimum of data to be collected for any outcome analysis, MED-B for a more detailed analysis, and MED-C data for a defined, but limited subgroup analysis. These basic principles still hold.

## “QMS 2.0”: next steps in the 1990s with mandatory reporting and first audits

When transplant numbers further increased and disease indications expanded, comparisons of individual series became more complex, despite the standardized outcome presentation. EBMT felt obliged to assure the correct presentation of successes and failures, and to maintain trust in the organization and the therapy. Three elements were introduced: a unique patient number (UPN), the activity survey, and first audits. EBMT teams adopted the standard format for a “UPN” for every individual patient. This principle did guarantee that all patients who had received a bone marrow or stem cell transplant were included in the center’s series. The day of the first infusion of the product was defined as day 0.

Beginning in 1990, and every year since, all teams were requested to report the numbers of their transplants in the preceding year by main disease category, donor type, and stem cell source, without reference to individual patients or outcome. Gathering of the “EBMT activity survey data” takes place separately and independently of the outcome data collection. This construct allows a direct quality control measure for individual centers by comparing the numbers of transplants performed, and the numbers of transplants reported to the disease specific data registries [21, 22].

In the 1990s, EBMT introduced systematic quality control audits for its member teams. One third of all teams were informed at the beginning of the year to be at risk for an audit. By lottery decision, ten teams were then selected. In a formal external audit, the UPN list of the center was compared with the center’s activity survey list. Furthermore, the data reported to the EBMT data-base of ten patients were checked for accuracy by their case histories [23].

## “QMS 3.0”: JACIE/FACT in the new century with a formal quality management system

### Development of JACIE/FACT

Several events fostered the introduction of the formal QMS “JACIE” at the end of last century. HSCT developed rapidly and somewhat erratically. A multitude of new disease categories were considered as indications; peripheral blood and cord blood were investigated as new stem cell sources. The growing registries of HLA-typed unrelated volunteers expanded the donor pool beyond matched family donors, and beyond borders. The sharp rise and fall of autologous HSCT for breast cancer called for better control of HSCT activity. Competent authorities worried about the risk of blood-borne viral diseases such as HIV, HTLV, or hepatitis when blood products or stem cells were exported or imported. Most countries in Europe asked for specific regulations of blood products, with varying priorities from one to the next. In this context, and believing that quality care can only be achieved if both clinical and laboratory issues are effectively addressed, EBMT took the opportunity to cooperate with the Foundation for Accreditation of Cellular Therapies (FACT) in the US. JACIE was formally established in 1999, leading to a hitherto unique collaboration on the jointly developed FACT–JACIE International Standards for Haematopoietic Cellular Therapy for Product Collection, Administration and Processing since 2002 [24–26].

The Standards are the cornerstone of the JACIE accreditation program, and currently apply to hematopoietic progenitor cells obtained from bone marrow, peripheral blood, or umbilical cord blood, and to hematopoietic cellular therapies. They are structured to align similar standards among the three primary functions within a transplant team: the clinical program, the collection facility, and the processing facility. This basic concept relies on defined responsibility, transparency, and reproducibility, with standard operating procedures for all clinical and laboratory steps. All actions are integrated into a continuous improvement, change control, and error management program. In parallel, EBMT has developed and continuously expanded recommendations and guidelines regarding all aspects of HSCT and cellular therapies [27]. FACT–JACIE standards have evolved over time, with scheduled review and revision based on the rapidly changing fields of HSCT and cellular therapy. They are based now on the 8th edition that extends to a broad range of cellular therapies, and searches for compatibility with the requirements for advanced therapeutic medicinal products (ATMPs) [27]. Currently, JACIE has become deeply rooted in the HSCT community with the FACT–JACIE standards a proven instrument to enable collaboration within and between centers, for donor product exchange, and have improved outcome of patients [28–30]. Several European competent authorities recognize JACIE standards for local or national authorization. To date, 367 teams in 29 countries have been accredited at least once after being audited.

### Current strengths and limitations

There is no question that FACT–JACIE standards have enhanced collaboration and improved outcome of patients. But novel steps have to be made for further improvement, to correct some major deficiencies in, and fine tuning of the current system. They relate to several independent domains. The wealth of transplant techniques and the increase in complex, novel cellular therapeutic products bring the current accreditation system to its limits. Requirements for the production of ATMPs as used for defined cellular products are high. The accreditation process for these procedures is complex, demanding, and relying on competent and trained inspectors. The latter became increasingly challenging, and, at several stages, impossible, since the start of the Covid-19 pandemic with its travel restrictions. For JACIE, the accreditation procedures currently followed are more difficult due to the many languages used in the different participating countries. Moving forward, there are also environmental benefits for limiting travel.

Today’s recommendations regarding choice of donor type, stem cell source, conditioning intensity and regimen, GvHD prevention method, and treatment of transplant complications have replaced the old “it can be done.” They follow the principles of evidence-based medicine. Still, they are too frequently based on sole expert opinion. Prospective randomized studies are often lacking. They are difficult to conduct in a multi-national Europe, with so many national regulatory processes, and without an equivalent to the Clinical Trials Network in the US. If done, they are often under-powered, or with errors in their design. Exploratory or confirmatory, retrospective, observational studies are frequently incomplete, their publications slow. This lack of high-level evidence fosters the trend to “individualize” transplant techniques. As a consequence, use of technologies varies substantially between centers and countries despite standardized recommendations [30–34]. This multitude of transplant techniques impedes comparisons, counteracts QMS strategies, and renders change control measures nearly impossible. This is a critical issue. Studies on large numbers of patients have failed to show that transplant techniques alter the inherent risk of pre-transplantation risk factors [35]. Not all techniques are equally efficient, equally cost-effective. Evidently, not all patients are treated with the optimal technique.

Lastly, and most importantly, the focus on the transplant procedure and its product fails to put the patient into the center of quality management. The goal of HSCT or any cellular therapy is clearly defined: it has to offer the best possible outcome for any individual patient with his or her disease, at his or her place with the given clinical conditions. Best outcome in this setting refers to overall survival, quality of life, and costs compared to any other therapy (Table 1). If not, HSCT should not be undertaken. This goal is missed with the current JACIE-FACT QMS approach. Patients in need of a transplant might not have received it; others might have been transplanted, already being cured from their disease. Whilst prognostication and decision making in each disease indication will be positively impacted by increasing use of personalized genomic testing-technologies of disease, patients and donors in routine practice, including whole genome sequencing, these aspects are not covered by the current system. The substantial differences within and between European centers and countries indicate that a substantial number of patients might not have been treated in an optimal way, and might not have been appropriately covered by the current system [22].

**Table 1:** Visions for the JACIE Quality Management System 4.0.

	QMS in general	JACIE 8th edition	JACIE “4.0”
*Goal*	“Product” quality	“Best transplant”	“Best treatment^a^”
*Principles*			
Responsibility	Who is doing what?	Defined by standards transplant team	Defined by standards treatment path
Transparency	Who has done what?	Data collection transplant team	Data collection treatment path
Reproducibility	Are the results consistent?	Data analysis transplants	Data analysis treatment path
*Change control*	Are goals defined and achieved?	Focus on transplanted patients	Focus on all patients with given disease
*Error management*	Basis for improvement	What went wrong, and why?	What went well, and why?
*Continuous improvement*	What has to be changed?	Focus on transplant techniques. How to improve the transplant?	Focus on treatment path. How to improve outcome for all patients?

^a^Best treatment defined as: the approach, which provides for a given patient with his/her disease, his/her history and current health status, his/her potential donor or advanced medical therapeutic product, his/her planned technology best possible outcome regarding long-term overall survival, quality of live and costs, compared to any other possible approach, including watch and wait or palliation, and based on formal evidence or on a prospective evidence generating study.

### New developments in QMS understanding

The classical QMS thinking dates back to early high-risk industries when nuclear power plants or aviation industry strived to identify structures and systems to ensure that “as few things as possible go wrong,” based on a “plan-do-check-act” principle of continuous quality and error management [36]. The systems were primarily based on a “safety first approach.” Such a view failed to consider that most systems function well, despite many errors, not “because people behave as they are supposed to, but because people can and do adjust what they do to match the conditions of work.” Safety management should therefore move to ensuring that “as many things as possible go right.” This Safety-II perspective assumes that “everyday performance variability provides the adaptations that are needed to respond to varying conditions, and hence that things go right. Humans are seen as the key resource for system flexibility and resilience.” In Safety-II perspectives, investigations and audits should help to understand how and why things usually go right, specifically under conditions where performance variability can become difficult or impossible to monitor. It forms the basis for explaining how things occasionally go wrong. Such a safety management’s principle is “to facilitate everyday work, to anticipate developments and events, and to maintain the adaptive capacity to respond effectively to the inevitable” [37, 38].

In a similar perspective, the Systems Change Working Group of the International Society for Quality in Health Care has recently summarized the current challenges regarding quality in health care [39, 40]. In a rapidly changing technical and fiscal system, classical QMS systems might run the risk to more hinder than promote safety of patients. New standards should rather “emphasize better coordination of care, address the entire health care journey, include patient-reported outcomes, reflect and predict technological changes and support new models of care.” Such new standards should “be less prescriptive, more flexible and incorporate new definitions of excellence and acceptability.” To achieve such goals, scientific organizations, governance bodies, external assessment agencies, and other authorities, as well as patients’ organizations, will be challenged to search for cooperation and collaboration [39, 40].

### Lessons from the Covid-19 pandemic

The Covid-19 pandemic has shown novel aspects for future directions in any field of medicine. Numbers of patients with Covid-19 infections, numbers of patients hospitalized, and numbers of patients dying from the disease have been collected in real time, by hospitals, states, countries, and on a global level [41]. Admittedly, it became possible out of an urgent need, and because some countries had pre-planned scenarios for a pandemic. Still, it shows that real-time assessment can be done. It should become possible as well for HSCT, by team, by country, and worldwide. The Covid-19 pandemic has also shown that correctly designed prospective randomized studies can be developed, conducted, and published in a very short time period. Some studies have shown that some approaches “believed to be helpful” did not hold their promise. These efforts should apply as well to HSCT; too frequently in HSCT, arguments prevail that randomization is not possible; the “new” HSCT approach is said to be too promising [42–45] encouraging teams to adopt these transplant techniques. Such arguments no longer hold, something reflected in the JACIE “self-check” certification exercise that was run during the second wave of the pandemic from August 2020 to March 2021.

## QMS 4.0: digitalization, the future

The future for JACIE should comprise deep changes on several independent but closely connected levels. It has to begin with a new definition of the QMS “product.” This will automatically change the focus of the basic QMS elements of responsibility, transparency, and reproducibility (Table 1). It will imply major reforms of organizational structures, and change the way of data collection, data analyses, and evidence generation.

### Product and service definition

Any QMS defines the structures leading to maintaining and continuously improving product and service quality [37, 38]. “Best transplant” or “best cellular therapy” no longer suffices in the field of HSCT. Any procedure, be it a stem cell transplant or a therapy with CAR-T cells, has to provide for each individual patient a “better outcome” regarding overall survival, quality of life, and costs compared to any alternative approach, including best supportive care or palliation. To do so, product definition has to change to best treatment as defined above and should refer to the complete treatment journey from diagnosis to long-term follow-up. As a consequence, patients’ outcomes in any comparison or benchmarking have to refer to all patients, transplanted or not; they have to include besides overall survival, quality of life, patient-reported outcome measures, and costs.

### Organizational structure

**Fig. 1:**
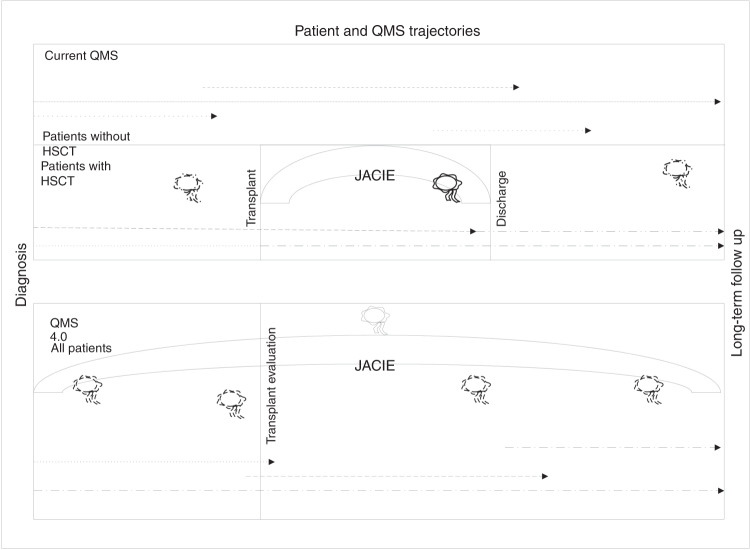
The place of JACIE QMS and audits today and in the future. Patient and QMS trajectories in the current QMS (upper part) and in a future QMS 4.0 (lower part). Arcs represent responsibilities of JACIE, flags audits.  Audits by JACIE or any accredited auditing agency.  Audits of the audits by specially trained JACIE inspectors.

The new QMS 4.0 will require substantial organizational changes, from JACIE, EBMT, and national organizations to the participating centers. QMS 4.0 will comprise standards and audits for teams as has been done so far, but include referring centers as well as centers responsible for continuous care to encompass patient trajectories from diagnosis to follow-up, on all levels (Fig. 1). It will be compatible, as already outlined in the 8th edition, with requirements for GMP and ATMP, and include standards and accreditation for regional and national organizations. In parallel, data collection and data transfer should become standardized, automated, and digitized on local, national, and global levels, along the whole health care journey. Scientific data collected by EBMT for the registries will have to be congruent with administrative data collected for competent authorities or insurances. They will have to be based on identical definitions, ideally compiled in a fully digitized way by compatible informatics systems.

It will require reorganizations by JACIE at all levels, in QMS education, inspectors training and audits. Audits will be more complex, inspectors’ requirements more challenging, training essential. Inspections of the complex cellular processing for ATMPs might be done in joint collaboration with the respective competent authorities, or being delegated to external professional auditors. In a similar way, audits of the patients’ paths before and after the transplant might be delegated to sister societies or professional auditors. As a consequence, JACIE might focus on inspector training requirements, with separate specific goals for internal or external inspectors. JACIE audits might then focus on the compatibility of standards along the treatment path as a whole.

### Risk directed, not risk adapted

As a consequence, the current “risk-adapted” strategies of HSCT become less relevant. They were based on earlier concepts that different conditioning regimens should be chosen for patients at high risk for relapse or high risk for non-relapse mortality; that different GvHD prevention methods should be chosen for patients at higher or lower risk for GvHD. Though highly plausible, retrospective studies so far failed to substantiate this hypothesis; transplant techniques do not alter the inherent risk of pre-transplantation risk factors [35, 46, 47]. Even more of a concern, the strategy might obscure the impact of HSCT on the total of patients with a given disease. Current risk-adapted strategies might select patients not needing a transplant, whilst losing patients in high need even before a transplant is considered. The implications for JACIE 4.0 appear clear: teams have to integrate in their QMS patients from diagnosis, to the transplant and beyond. They have to encompass referring centers regarding pre-HSCT algorithms, and follow-up.

### Harmonization

As a consequence, “individualized transplant techniques,” even well defined in local standards, no longer hold. Methods for donor type selection, and choice of stem cell source, conditioning, and GvHD prevention should become standardized. Harmonization between teams, cities, countries, and within EBMT will need a combined bottom-up/top-down strategy. If data fail to show an advantage for a specific form of transplant technique, the appropriate prospective studies should be conducted, or the respective technique should be abandoned in favor of the least expensive approach. EBMT with its working parties will be challenged to provide the appropriate framework, in cooperation with the national organizations and the respective national regulatory bodies.

### Cost considerations

Early in the history, JACIE was ironically called “Just Another Cost Increasing Exercise” [48]. Costs are too frequently forwarded as an argument why JACIE accreditation and conducting a QMS is not possible [49]. This view needs to be changed. HSCT is an expensive treatment. Costs for an autologous HSCT may vary around 50,000 Euros, for an allogeneic HSCT around 200,000 Euros [50, 51]. Without a QMS, without defined responsibilities, and without mandatory change control, it will remain unnoticed that some patients were erroneously transplanted, with the wrong donor, with insufficient conditioning or were given excessively expensive or futile drugs. This is wasted money, and specifically burdensome in times of limited resources. As an approximate estimate, total costs for the HSCT program in Europe 2019, with about 20,000 allogeneic and 30,000 autologous HSCT, were close to 5 billion Euros. There is room for a comprehensive QMS.

HSCT teams and the respective organizations should strive for the WHO concept that “data collection, data analysis, and quality control” are integral parts of any therapy, hence part of the costs for a transplant [52]. Competent authorities, health care insurance companies, and hospitals have to acknowledge these needs to the same extent and pay for data collection, data analysis, and quality care, as much as they do for specified drugs, hospitalizations, or out-patient costs [53].

## Conclusions

We have outlined the evolution of “quality concepts” in HSCT over time, from the very beginning to today’s formal JACIE QMS. We have looked at the individual steps in their respective time frames, and summarized current strengths and deficiencies. The outlook appears clear. It is not possible to go back; but future JACIE standards will require some fundamental changes. Still based on transparency, responsibility, reproducibility, and accountability, future standards will have to put the “primary product and the primary process” into the center of the QMS. This goal is defined: the patient. The basis of any future QMS must be to help achieve the best outcome for individual patients as defined above, by whatever treatment strategy, including, but not exclusively, cellular therapies, and at the most efficient costs, along the whole patient journey. All steps have to be based on stringent evidence. QMS concepts have to focus less on avoiding failures, but should strive to achieve good outcome for the most, and support patients, families, and health care personal in their efforts. To achieve this goal, the modern instruments of Medicine 4.0 have to be integrated, at the local, national, and global level, in real time, from diagnosis to continuous follow-up. Evidently, such a concept will further restrict the autonomy of individual transplant physicians and individual transplant centers. Uncontrolled small-scale pilot studies will disappear, and be replaced by clearly defined prospective randomized Phase I/II/II studies. They will assess the role of individual transplant technologies, as well as the role of the transplant or cellular therapy itself. The vast differences in the use of transplant technologies within and between European countries will fade.

This concept 4.0 will not permit to go on with business as usual. It will oblige EBMT, national organizations, and individual teams to search for intensified cooperation internally and externally. It will need recognition that such a QMS is nothing “nice to have,” but essential from the very beginning. The concept 4.0 will have to include cooperation with competent authorities, health care providers, insurances, as well as information technologies, private and public research organizations, and pharmaceutical companies. It will have to rely in some parts of external auditors and regulators. It will require a tremendous effort to bring this concept forward, but it can be done. This transformation will be challenging, and will not be achieved in one step, but should start now. Awareness of the need is the most essential step. If successful, HSCT could again serve as a role model for hemato-oncology and life-threatening blood disorders in general. To reach this goal, the process has to start with harmonization within and between organizations at institutional and national levels, in a combined bottom-up top-down process. Acceleration in IT systems and other modifications to working practices during the pandemic should facilitate this development of quality management to the next stage.

## References

[1] Jacobson LO, Marks EK, Gaston EO, Robson M, Zirkle RE (1949). The role of the spleen in radiation injury. Proc Soc Exp Biol Med..

[2] Lorenz E, Uphoff D, Reid TR, Shelton E (1951). Modification of irradiation injury in mice and Guinea pigs by bone marrow injections. J Natl Cancer Inst..

[3] Ford CE, Hamerton JL, Barnes DW, Loutit JF (1956). Cytological identification of radiation-chimaeras. Nature..

[4] Mathé G, Jammet H, Pendic B, Schwarzenberg L, Duplan JF, Maupin B (1959). Transfusions and grafts of homologous bone marrow in humans after accidental high dosage irradiation. Rev Fr Etud Clin Biol..

[5] Thomas ED. A history of allogeneic hematopoietic cell transplantation. In: Appelbaum FR, Forman SJ, Negrin RS, Blume KG, editors. Thomas’ hematopoietic cell transplantation. Chichester: Wiley; 2007. p. 3–7.

[6] Gratwohl A, Mohty M, Apperley J. The EBMT: history, present, future. In: Carreras E, Dufour C, Mohty M, Kröger N, editors. The EBMT handbook: hematopoietic stem cell transplantation and cellular therapies. 7th ed. Cham (CH): Springer; 2019. Chapter 2.

[7] Thomas ED, Lochte HL, Lu WC, Ferrebee JW (1957). Intravenous infusion of bone marrow in patients receiving radiation and chemotherapy. N Engl J Med..

[8] Thomas ED, Storb R, Clift RA, Fefer A, Johnson L, Neiman PE (1975). Bone-marrow transplantation (second of two parts). N Engl J Med..

[9] Copelan EA (2006). Hematopoietic stem cell transplantation. N Engl J Med..

[10] Gratwohl A, Baldomero H, Aljurf M, Pasquini MC, Bouzas LF, Yoshimi A (2010). Hematopoietic stem cell transplantation: a global perspective. JAMA..

[11] Sharma A, Badawy SM, Suelzer EM, Murthy HS, Prasad P, Eissa H (2021). Systematic reviews in hematopoietic cell transplantation and cellular therapy: considerations and guidance from the American Society for Transplantation and Cellular Therapy, European Society for Blood and Marrow Transplantation, and Center for International Blood and Marrow Transplant Research Late Effects and Quality of Life Working Committee. Transpl Cell Ther..

[12] Spier F, editor. Big history and the future of humanity. Chichester: Wiley; 2015.

[13] Global Future Council on the Future of Health and Healthcare 2016–2018 of the World Economic Forum. Health and Healthcare in the Fourth Industrial Revolution. 2019. http://www3.weforum.org/docs/WEF__Shaping_the_Future_of_Health_Council_Report.pdf. Assessed 30 Aug 2021.

[14] Schwab K. The Fourth Industrial Revolution | Foreign Affairs. 2015. Assessed 30 Aug 2021.

[15] Hood L, Balling R, Auffray C (2012). Revolutionizing medicine in the 21st century through systems approaches. Biotechnol J..

[16] Chen C, Loh EW, Kuo KN, Tam KW (2020). The times they are a-Changin’ – Healthcare 4.0 is coming!. J Med Syst.

[17] Javaid M, Haleem A (2019). Industry 4.0 applications in medical field: a brief review. Curr Med Res Pract.

[18] Verghese A, Shah NH, Harrington RA (2018). What this computer needs is a physician humanism and artificial intelligence. JAMA..

[19] Bortin MM (1970). A compendium of reported human bone marrow transplants. Transplantation..

[20] Clift R, Goldman J, Gratwohl A, Horowitz M (1989). Proposals for standardized reporting of results of bone marrow transplantation for leukaemia. Bone Marrow Transplant.

[21] Gratwohl A (1991). Bone marrow transplantation activity in Europe 1990. European Group for Bone Marrow Transplantation (EBMT). Bone Marrow Transplant.

[22] Passweg JR, Baldomero H, Chabannon C, Basak GW, de la Camara R, Corbacioglu S (2021). European Society for Blood and Marrow Transplantation (EBMT). Hematopoietic cell transplantation and cellular therapy survey of the EBMT: monitoring of activities and trends over 30 years. Bone Marrow Transplant.

[23] Gratwohl A. Organizational aspects. In: Gluckman E, Apperley J, Gratwohl A, editors. The EBMT handbook blood and marrow transplantation. Paris: European School of Haematology; 1998. p. 10–27.

[24] Serke S, Johnsen HE (2001). A European reference protocol for quality assessment and clinical validation of autologous haematopoietic blood progenitor and stem cell grafts. Bone Marrow Transplant.

[25] Warkentin PI; Foundation for the Accreditation of Cellular Therapy. Voluntary accreditation of cellular therapies: Foundation for the Accreditation of Cellular Therapy (FACT). Cytotherapy. 2003;5:299–305. 10.1080/14653240310002298 12944235

[26] Kvalheim G, Gratwohl A, Urbano-Ispizua A (2003). JACIE national representatives. JACIE accreditation in Europe moves ahead. Cytotherapy..

[27] EBMT. 8th edition of the fact-jacie standards. 2021. https://www.ebmt.org/8th-edition-fact-jacie-standards. Assessed 30 Aug 2021.

[28] Gratwohl A, Brand R, Niederwieser D, Baldomero H, Chabannon C, Cornelissen J (2011). Introduction of a quality management system and outcome after hematopoietic stem-cell transplantation. J Clin Oncol..

[29] Snowden JA, McGrath E, Duarte RF, Saccardi R, Orchard K, Worel N, et al. JACIE accreditation for blood and marrow transplantation: past, present and future directions of an international model for healthcare quality improvement. Bone Marrow Transplant. 2017;52:1367–71. 10.1038/bmt.2017.54 PMC5629362 28346416

[30] Snowden JA, Saccardi R, Orchard K, Ljungman P, Duarte RF, Labopin M (2020). Benchmarking of survival outcomes following haematopoietic stem cell transplantation: A review of existing processes and the introduction of an international system from the European Society for Blood and Marrow Transplantation (EBMT) and the Joint Accreditation Committee of ISCT and EBMT (JACIE). Bone Marrow Transplant.

[31] Anthias C, Apperley J, Bloor A, Byrne J, Collin M, Crawley C (2020). Reducing the diversity of allogeneic transplant protocols in the UK through a BSBMT Anthony Nolan Protocol Harmonization Initiative. Bone Marrow Transplant..

[32] Penack O, Marchetti M, Ruutu T, Aljurf M, Bacigalupo A, Bonifazi F (2020). Prophylaxis and management of graft versus host disease after stem-cell transplantation for haematological malignancies: updated consensus recommendations of the European Society for Blood and Marrow Transplantation. Lancet Haematol..

[33] Short NJ, Zhou S, Fu C, Berry DA, Walter RB, Freeman SD (2020). Association of measurable residual disease with survival outcomes in patients with acute myeloid leukemia: a systematic review and meta-analysis. JAMA Oncol..

[34] Gratwohl A (2020). Ruxolitinib for acute graft-versus-host disease. N Engl J Med..

[35] Gratwohl A, Passweg J, Baldomero H, Orchard K, Kröger N, Snowden JA (2021). Joint Accreditation Committee (JACIE) of the International Society for Cellular Therapy Europe (ISCT), the European Society for Blood, Marrow Transplantation (EBMT) Conditioning intensity before allogeneic haematopoietic stem cell transplantation: a quality control audit. Br J Haematol..

[36] Quality management – Wikipedia. Assessed 30 Aug 2021.

[37] What is a Quality Management System (QMS)? | ASQ. Assessed 30 Aug 2021.

[38] EFQM Model | EFQM. Assessed 30 Aug 2021.

[39] Braithwaite J, Vincent C, Nicklin W, Amalberti R (2019). Coping with more people with more illness. Part 2: new generation of standards for enabling healthcare system transformation and sustainability. Int J Qual Health Care.

[40] Braithwaite J, Wears RL, Hollnagel E (2015). Resilient health care: turning patient safety on its head. Int J Qual Health Care.

[41] Home – Johns Hopkins Coronavirus Resource Center (jhu.edu). Assessed 30 Aug 2021.

[42] Kalil AC (2020). Treating COVID-19-off-label drug use, compassionate use, and randomized clinical trials during pandemics. JAMA.

[43] McDermott MM, Newman AB (2020). Preserving clinical trial integrity during the coronavirus pandemic. JAMA..

[44] Spinner CD, Gottlieb RL, Criner GJ, Arribas López JR, Cattelan AM, Soriano Viladomiu A (2020). Effect of remdesivir vs standard care on clinical status at 11 days in patients with moderate COVID-19: a randomized clinical trial. JAMA..

[45] WHO Ad Hoc Expert Group on the Next Steps for Covid-19 Vaccine Evaluation. (2021). Perspective. Placebo-controlled trials of Covid-19 vaccines—why we still need them. N Engl J Med..

[46] Gratwohl A, Sureda A, Cornelissen J, Apperley J, Dreger P, Duarte R (2017). Alloreactivity: the Janus-face of hematopoietic stem cell transplantation. Leukemia..

[47] Gratwohl A, Duarte R, Snowden JA, van Biezen A, Baldomero H, Apperley J (2019). Joint Accreditation Committee JACIE. Pre-transplantation risks and transplant-techniques in haematopoietic stem cell transplantation for acute leukaemia. EClinicalMedicine.

[48] Apperley J (2004). Just another cost increasing exercise (JACIE)?. Bone Marrow Transplant.

[49] Zahnd D, Leibundgut K, Zenhäusern R, Pabst T, Fontana S, Schneider R (2004). Implementation of the JACIE standards for a haematopoietic progenitor cell transplantation programme: a cost analysis. Bone Marrow Transplant.

[50] Svahn BM, Remberger M, Alvin O, Karlsson H, Ringdén O (2012). Increased costs after allogeneic haematopoietic SCT are associated with major complications and re-transplantation. Bone Marrow Transplant.

[51] Perales MA, Bonafede M, Cai Q, Garfin PM, McMorrow D, Josephson NC (2017). Real-world economic burden associated with transplantation-related complications. Biol Blood Marrow Transpl.

[52] World Health Organization. (2010). WHO guiding principles on human cell, tissue and organ transplantation. Transplantation..

[53] Stafinski T, McCabe CJ, Menon D (2010). Funding the unfundable: mechanisms for managing uncertainty in decisions on the introduction of new and innovative technologies into healthcare systems. Pharmacoeconomics.

